# Mapping Peptidergic Cells in *Drosophila*: Where DIMM Fits In

**DOI:** 10.1371/journal.pone.0001896

**Published:** 2008-03-26

**Authors:** Dongkook Park, Jan A. Veenstra, Jae H. Park, Paul H. Taghert

**Affiliations:** 1 Department of Anatomy and Neurobiology, Washington University School of Medicine, Saint Louis, Missouri, United States of America; 2 CNIC UMR 5228 CNRS, Université Bordeaux I, Talence, France; 3 Department of Biochemistry and Cellular and Molecular Biology, University of Tennessee–Knoxville, Knoxville, Tennessee, United States of America; Columbia University, United States of America

## Abstract

The bHLH transcription factor DIMMED has been associated with the differentiation of peptidergic cells in *Drosophila*. However, whether all *Drosophila* peptidergic cells express DIMM, and the extent to which all DIMM cells are peptidergic, have not been determined. To address these issues, we have mapped DIMM expression in the central nervous system (CNS) and periphery in the late larval stage *Drosophila*. At 100 hr after egg-laying, DIMM immunosignals are largely congruent with a *dimm*-promoter reporter (*c929*-GAL4) and they present a stereotyped pattern of 306 CNS cells and 52 peripheral cells. We assigned positional values for all DIMM CNS cells with respect to reference gene expression patterns, or to patterns of secondary neuroblast lineages. We could assign provisional peptide identities to 68% of DIMM-expressing CNS cells (207/306) and to 73% of DIMM-expressing peripheral cells (38/52) using a panel of 24 markers for *Drosophila* neuropeptide genes. Furthermore, we found that DIMM co-expression was a prevalent feature within single neuropeptide marker expression patterns. Of the 24 CNS neuropeptide gene patterns we studied, six patterns are >90% DIMM-positive, while 16 of 22 patterns are >40% DIMM-positive. Thus most or all DIMM cells in *Drosophila* appear to be peptidergic, and many but not all peptidergic cells express DIMM. The co-incidence of DIMM-expression among peptidergic cells is best explained by a hypothesis that DIMM promotes a specific neurosecretory phenotype we term LEAP. LEAP denotes Large cells that display Episodic release of Amidated Peptides.

## Introduction

Neuropeptides were first studied as chemical messengers secreted by hormone-producing neurons[1]. For example, magnocellular neurosecretory neurons synthesize and release vasopressin and oxytocin [2]. Neuropeptides are also secreted by conventional neurons as co-transmitters with small, fast-acting chemicals [3]. For example, mammalian skeletal motorneurons release various neuropeptides along with acetylcholine[4]. Likewise, single modulatory interneurons in crustacea release neuropeptides along with GABA to affect distinct responses in neuronal function[5].

*Drosophila* genetics provides useful research tools to investigate the physiology of peptidergic and neuroendocrine systems [6]–[13]. Annotations of the *Drosophila* genome indicate it contains roughly 30 neuropeptide-encoding genes and roughly 45 genes encoding G protein-coupled neuropeptide receptors [14]–[17]. In parallel efforts, biochemical surveys of *Drosophila* have begun to systematically analyze and catalogue the *Drosophila* peptidome [18]–[22]. Most recently, Wegener and colleagues have begun to define the neuroarchitecture of peptidergic projections within the CNS to define the morphological rules by which peptidergic neurons receive and send information [23]. The present work is a contribution in the same vein: we attempt to provide an overall map for an important developmental regulator of *Drosophila* peptidergic cells, the basic helix loop helix (bHLH) transcription factor DIMMED.

The precise profiles of transmitters and neuropeptides that are produced by different cell types as a function of their positions along the neuraxis are highly reproducible. Many cell-intrinsic regulatory mechanisms that help establish specific transmitter phenotypes have been discovered (e.g., [24], [25]). However, the relevant developmental mechanisms that underlie peptidergic cell properties, especially those of neurosecretory peptidergic neurons, remain poorly understood. Transcription factors such as Mash1, Otp, Brn2, Sim1 and Sim2 are known to regulate the early differentiation of hypothalamic neuroendocrine centers by their expression in neuronal progenitors and in pre-migratory neurons[26]–[32]. However little is known about the intrinsic regulatory factors that directly organize maturation of peptidergic cellular properties.

DIMMED protein (DIMM) has a limited expression pattern within the CNS and periphery and, for the most part, first appears within cells that have recently become post-mitotic [33], [34]. There are a few examples known of neurons that become post-mitotic in the embryo, but which then delay differentiation until metamorphosis: in those cases, DIMM expression is likewise delayed[35]. DIMM is a member of the NeuroD family of bHLH transcription factors and its mammalian sequence orthologue is Mist1[36]. Mist1 is required for normal differentiation of serous exocrine cells[37], [38]. For example, in Chief Cells of the stomach, Mist1 is dispensible for cell survival, but is needed to complete cellular trans-differentiation to display a robust zymogenic phenotype that includes a highly active, regulated secretory pathway [39]. In the fly, DIMM is a transcription factor whose direct targets include *PHM* [40]–this gene encodes the enzyme regulating the rate-limiting step for C-terminal neuropeptide amidation [9], [41]. Amidation is a specific and critical post-translational modification displayed by the vast majority of *Drosophila* neuropeptides[9], [14]. Mammalian Mist1 trans-activates *Drosophila PHM* in cell culture and like DIMM, it can drive ectopic expression of *PHM* in non-peptidergic neurons of the fly in a transgenic model[40]. Initial descriptions in *Drosophila* have linked DIMM expression with peptidergic neurons and with peripheral endocrine cells[33]–[35], [40], [42], [43], though it is clearly not tied to any single neuropeptide or peptide hormone. Miguel-Aliaga *et al*. [44] surveyed DIMM-positive neurons in the Stage 17 embryo ventral nerve cord and could ascribe several different molecular markers to a large subset of them. However, there has not been a comprehensive effort to map DIMM expression, or to evaluate the degree to which its correlation with peptidergic cells is partial or complete.

Here we map and identify nearly all 306 DIMM-positive cells in the larval central nervous system. Furthermore, we use a large panel of peptide antibodies and gene reporters to survey DIMM expression in the context of *Drosophila* peptidergic systems. Our observations reveal a substantial correlation of DIMM expression with peptidergic phenotypes. Most or all DIMM cells are peptidergic, but importantly, not all peptidergic cells are DIMM-positive. We observe that DIMM is generally expressed by those peptidergic cells that display the highest level of steady-state secretory activity and which extend longer and more complex neuronal processes–we define these as Neurosecretory Neurons and give them the acronym LEAP, which stands for Large cells that display Episodic release of Amidated Peptides. We argue that at a molecular level, DIMM concerns secretory peptides that are amidated, and at a cellular level, DIMM concerns peptidergic neurons which are Neurosecretory. We conclude that DIMM plays a dedicated role to promote the differentiation of most of the principle Neurosecretory (including neuroendocrine) cells in the fly. A corollary to this conclusion is that in *Drosophila,* there exist alternative regulatory pathways for the control of peptidergic phenotypes in non-DIMM cells. Furthermore, we propose that different peptidergic cells can be usefully described by their divergent regulatory cascades, of which DIMM controls one.

## Results

### DIMM protein expression closely follows the pattern of *c929*-GAL4

**Figure 1:**
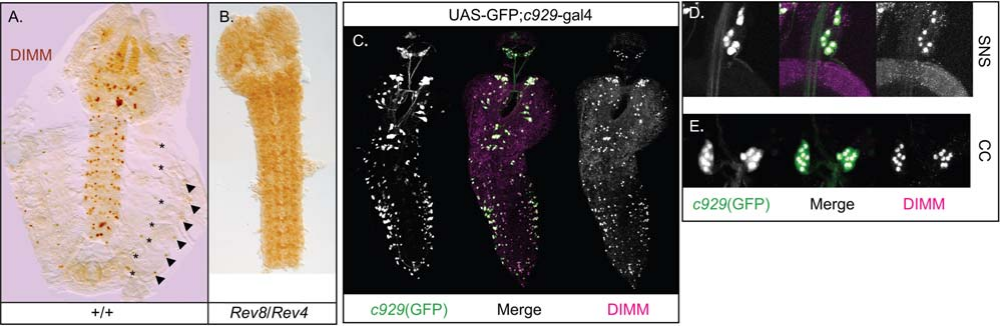
DIMM immunostaining and its correspondence to *c929*-GAL4 expression. A) Anti-DIMM staining in St 17 wild type embryo and (B) in a *dimm* mutant (*Rev8*/*Rev4*). Asterisks mark the peripheral LBD neuron; Arrowheads mark positions of the Inka cell. C) *c929*(*dimm*) signals are highly congruent with DIMM antibody staining. Arrows mark the few DIMM cells that are not *c929*-positive. DIMM-positive neurons are also *c929*-positive in (D) the stomatogastric nervous system (SNS), and (E) in the sixteen cells of corpora cardiaca (CC) within the Ring Gland.

The P element *c929*-gal4 is inserted within the gene *cryptocephal* and lies ∼13kb upstream of *dimm* (Hewes *et al.,* 2003). We previously showed that the expression of *crc* mRNA is largely ubiquitous in the larval central nervous system (CNS), while that of *dimm* paralleled the *c929*-GAL4 pattern. To investigate this correspondence at the protein level and with better resolution, we used antibodies directed against the C-terminal domain of the DIMM protein as described by Allan *et al.* [34]. Here we show in the 3^rd^ larval instar, most *c929*-positive cells represent DIMM-positive cells **(** **Figure 1**).

In the wild type embryo, DIMM immunosignals appeared transiently in broad domains of the ectoderm, beginning at St 11 and disappeared by St 14. DIMM protein then appeared in a stable and reproducible pattern in several hundred cells of the CNS and periphery (**Figure 1A**). In the periphery, anti-DIMM labeled the dorsal pharyngeal muscle (not shown), the lateral bipolar dendrite neurons[45], cells associated with the corpora cardiaca of the Ring Gland, the trachea-associated Inka endocrine cells, and cells associated with the developing heart (not shown). This pattern of expression persisted throughout the larval stages, with only minor changes. At a sub-cellular level, signals were concentrated in the nucleus and also in small cytoplasmic inclusions *(visible in later figures)*; the inclusions were more prevalent in younger specimens such as embryos, 1^st^ and 2^nd^ instar larvae, and were generally absent in feeding stage, 3^rd^ instar larvae (data not shown; see also Supplemental Information). All nuclear signals appeared specific because they were absent in *dimm* mutants (**Figure 1B**), while cytoplasmic inclusions were non-specific-present regardless of the *dimm* genotype. Among the stained nuclei, we observed different levels of staining intensity: in this effort to map DIMM expression, we focused on strong signals and did not score low-level DIMM expression (**Figure S1**).

In the larval CNS, greater than 90% of DIMM-positive cells expressed *c929-*GAL4, and virtually all GAL4-positive neurons expressed DIMM (**Figure 1C** and **Figure S1**). The *c929*-GAL4 pattern includes some surface glia in the adult brain (data not shown); in the larval CNS, no surface glia were DIMM immuno-positive. Next, we asked whether peripheral neuroendocrine cells that express *c929*-GAL4 also express DIMM. We found DIMM co-localization in all the major endocrine and neuroendocrine locations including, in the segmental lateral bipolar dendrite neuron (LBD) of the peripheral nervous system (**Figure 1A**), in seven cells of the stomatogastric nervous system (SNS) (**Figure 1D**), in all 16 neuroendocrine cells within the corpora cardiaca (CC) of the Ring Gland (**Figure 1E**), and in the 14 endocrine Inka cells associated with the tracheal system (**Figure 1A**). However, several cells and tissues that normally express *c929*-GAL4 did not display detectable DIMM immunosignals: the salivary gland, fat body, and tracheal cells.

In summary, we showed that the anti-DIMM antibody is genetically-specific and that its expression pattern that is largely congruent with that of *c929*-GAL4. In the following sections we examine the pattern of DIMM expression in greater detail.

### A map of DIMM-positive neurons in the larval CNS

**Figure 2:**
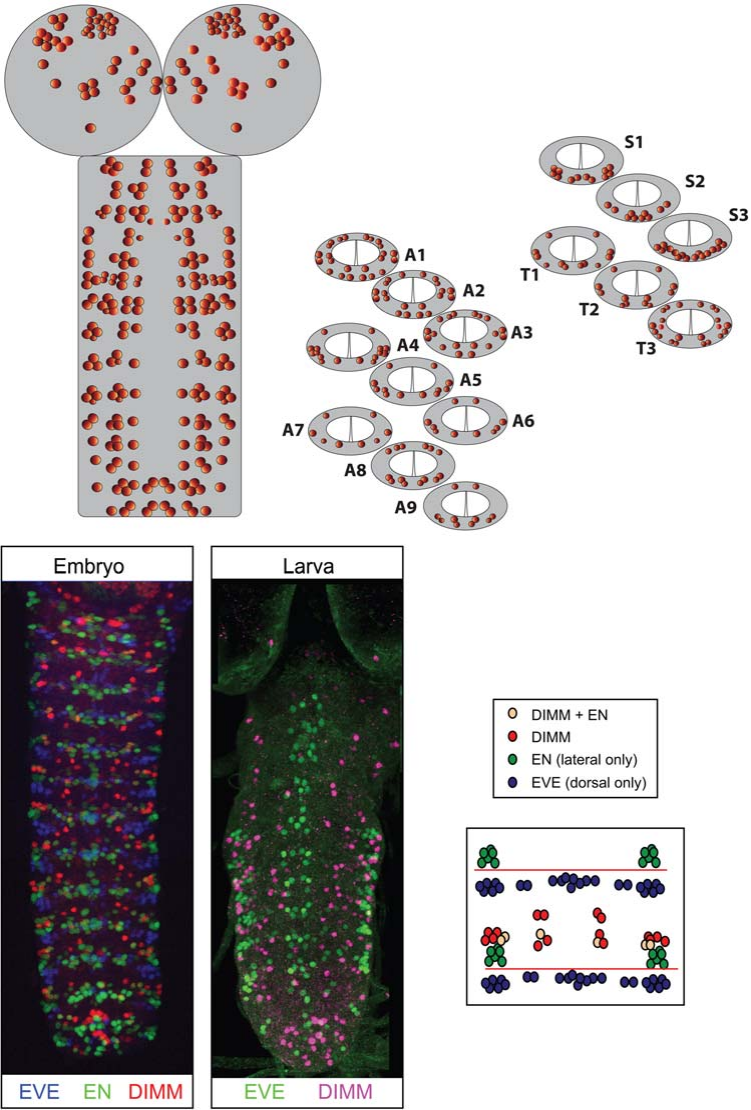
A map of DIMM-expressing neurons in the 100 hr AEL larval CNS. Top: schematic of the CNS along with cross-sections of each segmental neuromere to illustrate dorsal-ventral positions of DIMM-expressing neurons. Details on positions of individual DIMM-positive neurons in the brain hemispheres are given in later figures. Bottom: use of EVE and EN expression patterns to assign DIMM neurons to individual segments. Left: triple-labeled St. 17 embryonic CNS stained for EVE (blue) EN (green) and DIMM (red); middle: double-labeled 100 hr AEL larval CNS stained for EVE (green) and DIMM (red); right: schematic of an idealized abdominal segment showing the interpretation used to assign segmental values to DIMM neurons in the VNC.

We studied DIMM expression in late stage embryonic CNS (St 17) and in the CNS of feeding 3^rd^ instar larvae that were approximately 100 hr after egg laying (AEL). The earlier stage produced clear DIMM expression signals but poorly developed patterns of neuropeptide expression. The latter stage produced robust neuropeptide expression as well as strong and maintained DIMM expression. The DIMM expression pattern was basically constant between the two developmental stages, and a simplified overview is illustrated in **Figure 2A**. It includes 306 cells that are distributed throughout the rostral-caudal axis of the CNS: a total of 45 DIMM cells are found at different, though reproducible locations within each brain hemisphere, and 8–22 DIMM cells are observed per segmental neuromere, for a total of 216 in the VNC.

#### DIMM cells in the larval VNC

In order to provide a segmentally-accurate map of DIMM cells, we employed the well-described *even-skipped* (EVE) and *engrailed* (EN) markers of segmental identity [46]. Triple-labels of EVE/EN and DIMM were performed on late stage (St. 17) embryos (**Figure 2B**) and double-labels (EVE/DIMM) on 100 hr AEL larval nervous systems (**Figure 2C**). The developmental stability of the DIMM expression map allowed us to assign provisional segmental identities to larval DIMM cells based on similarity to their inferred positions in the embryonic CNS (**Figure 2D**). Of these, one lateral DIMM neuron per abdominal hemisegment was EVE-positive in the larval, but not the embryonic CNS; three DIMM cells were EN-positive per thoracic and abdominal hemisegments in both embryonic and larval CNS. The EL group of EVE neurons lies immediately posterior to the segmental boundary and thus roughly marks the anterior domain of a neuromere [46]. Likewise, the PL group of EN neurons provides a rough position for the posterior aspect of the neuromere [47]. Our interpretation places DIMM cells essentially between the EVE and EN indicators of segmental boundaries (**Figure 2D**). On this basis, we assigned provisional segmental identity to each DIMM-positive neuron in the larval ventral nerve cord (as described more fully in later figures).

#### DIMM neurons in the larval brain

**Figure 3:**
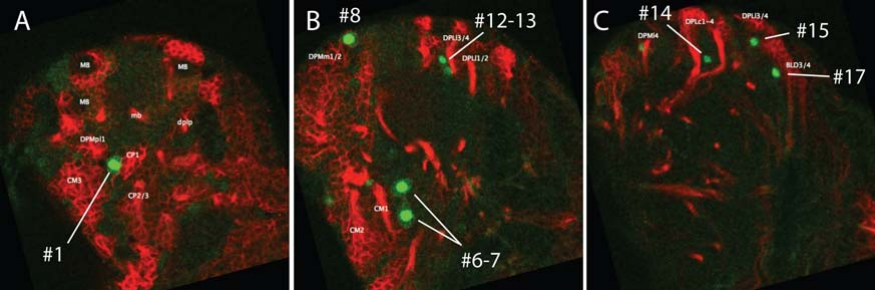
Mapping positions of DIMM-positive neurons in the larval brain hemispheres. (A–C) Representative single confocal scans of a 100 hr AEL larval brain hemisphere that was double-labeled for MAb BP106 (anti-neurotactin) and anti-DIMM (green). (A) In the dorsal aspect, Cell #1 (MP0) lies adjacent to the CP1 secondary neuroblast lineage. (B) at a mid-dorsal position, Cell #8 is adjacent to DPMm1/2, Cells 12 and 13 are adjacent to DPLl3/4 and cells 6–7 are adjacent to CM1; (C) more ventral, Cell #14 is found close to DPLc1-4, Cell #15 close to DPL3/4 and Cell #17 close to BLD3/4. See Table 1 for the complete listing of results from this analysis.

Pereanu and Hartenstein (2006) established a 3D atlas of the pattern of identified neuronal lineages of the larval brain based on the anti-neurotactin (MAb BP106) staining pattern. This atlas serves as a framework on which gene expression patterns may be positioned and then compared in a consistent and systematic fashion. Thus, to provide a positional reference system for DIMM-positive brain cells, we determined the proximity of individual DIMM cells to identified secondary neuroblast lineages[48]. **Figures 3B, C and D** illustrate DIMM/neurotactin double staining images at different dorsal-ventral levels. ∼100 neuroblast lineages can be distinguished in the 3^rd^ instar larval brain, and we were able to assign relative positions to 43 of the 45 DIMM-positive brain cells (**Table 1**). One or two in the ventral brain were seen only variably and hence could not be accurately scored. Each of the 45 cells was assigned an arbitrary numerical identity and, where possible, the closest neuronal lineage(s) were defined from a set of three specimens.

**Table 1:** Positions of DIMM-positive cells in the brain.

Cell#	Cell Name	NP Marker	Secondary Lineage(s)
1	MP3	Unknown	CP1, CM1
2–3	MP 4–5	CCAP	DPMpl1, CM3
4–5	MP1	*DSK*-GAL4/AstC	CM2, CM3
6–7	MP2	sNPF/DMS	CP1, CM1
8	NPF-M	*NPF*-GAL4	DPM1/2, DPMl3
9–11	PL 1–3	DMS/ ITP	DPLl3/4, BLD5
12–14	PL 4–6	Crz	DPLl1/2, DPL3/4, DPMl4
15	PL 7	DMS/ ITP	DPLl3/4, BLD5
16	PL 8	Unknown	DPLl3/4, BLD5
17	PL 9	Leucokinin	BLD3/4, BLD1/2
18	PL10 (NPF-L)	*NPF*-GAL4	BLD3/4, BLD1/2
19–25	PI 1–7	dILP/*DSK*-GAL4	PI
26–27	PI 8–9	SIFa	PI
28–30	PI 10–12	DH 44	PI
31–32	PI 13–14	DMS	PI
33–34	PI 15–16	Unknown	PI
35	VP 1	Ast-A	BAlp1/2/3
36	VA 1	Unknown	Not assigned
37	VA 2	EH	DAMd2/3, DALcm1
38	VA 3	DTK/Ast-B	DAMd1
39	VA 4	Unknown	DAMd2/3, DALcm1
40–41	VP 2–3	Unknown	damv
42	VP 4	Unknown	Bamv2, DALcm2
43	VP 5	Unknown	Bamd1
44–45	VP 6	Unknown	Not assigned

Cells not assigned to Secondary lineages were either not reproducible in position or variably-stained.

Notes: In the cell group 40–43, one of the cells is DH 31-positive, but we did not determine which. Abbreviations: NP: neuropeptide; MP: medial protocerebral; PI: Pars Intercerebralis; PL: Pars Lateralis; VA: ventral anterior; VP: ventral posterior;

### Mapping the peptide identities of DIMM-expressing cells

Previous studies have shown that DIMM regulates cellular phenotypes in diverse peptidergic neurons of *Drosophila* [33], [34], [40]. For example, the identified Tv neurons of thoracic segments that express *dFMRFa* and the identifiable *leukokinin*-expressing neurons of the abdominal segments all display strong regulation by DIMM [33], [34]. Likewise steady-state levels of the neuropeptide processing enzyme PHM (peptidylglycine alpha-hydroxylating monooxygenase) are sensitive to loss of DIMM[33], [40]. Moreover, gain-of-function analysis shows that DIMM can confer strong PHM expression, and thus a peptidergic phenotype, onto all neurons of the CNS [34]. These studies strongly suggest that DIMM is highly correlated with, and linked to, mechanisms of peptidergic differentiation. Therefore, we asked-how many of the 306 DIMM-expressing cells in the CNS can be related to markers of known neuropeptides? Also we wondered if (or to what extent) the set of DIMM cells represents a population that is homogenous and dedicated to peptidergic cell function.

**Figure 4:**
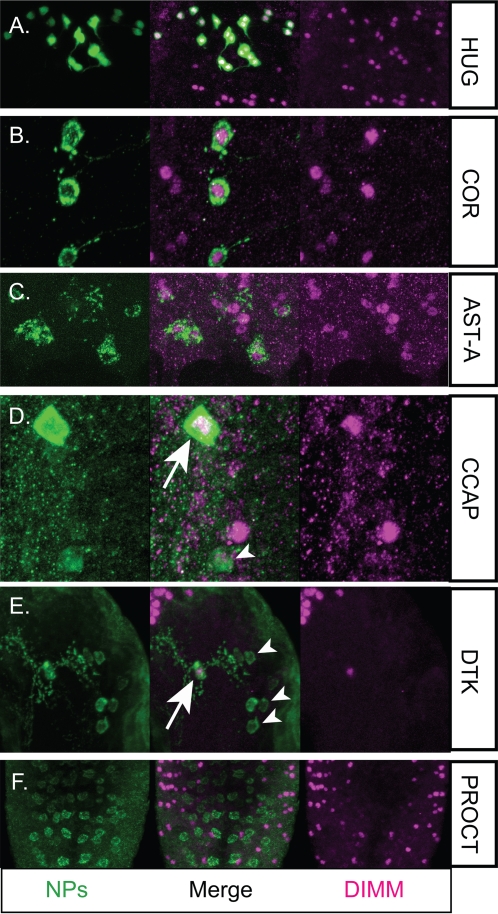
Examples of double-antibody stains performed in the CNS of 100 hr AEL larvae to compare DIMM immunosignals with those for markers of 24 different peptidergic systems. Table S1 provides the summary of numerical results from this analysis; Table 2 lists the markers used. Figure 5, 6, 7, 8, 9 and 10 provide more details of DIMM/peptide marker overlap for each CNS region and for each peptide marker. Overlap of DIMM and different peptides varies from complete to virtually none. (A) An example of a peptide system that exhibits complete overlap with DIMM: Hugin-YFP-neurons in S1 and S2 are all strongly DIMM-positive. (B) Of several COR-immunopositive neurons in the CNS, several are DIMM positive. (C–E). Examples of peptide systems that exhibit partial overlap with DIMM. (C) The most strongly stained Ast-A-positive neurons are also DIMM-positive. (D) Likewise, the most strongly stained CCAP-expressing neurons are DIMM-positive (arrow), while the weakly stained cell is DIMM-negative (arrowhead). (E) The dTK system shows only a single DIMM-positive cell (arrow) among many DIMM-negative dTK-expressing cells (arrowheads): it is the largest and most strongly-stained. (F) An example of little or no overlap with DIMM: anti-proctolin antibodies label several hundred neurons in the CNS, of which only one cell type–the Ap-let neuron [35] is weakly stained by proctolin antibodies but is strongly DIMM-positive. NPs: neuropeptides.

**Figure 4** shows examples of double-staining for DIMM and various neuropeptide markers. As described in a later section, we observed partial to complete overlap of DIMM with various neuropeptide markers. In total, we examined more than 24 neuropeptide markers (see **Tables 2** **and** **3**) for potential co-localization with DIMM in the 100 hr AEL larval CNS. The **Tables** list markers for 26 genes, but we do not include AKH or ETH markers in this section, as they are only expressed by peripheral endocrine/neuroendocrine cells. In fact all *ETH*- and *AKH*-expressing cells are also DIMM-positive (**Figure 1**, **Figure S2** and data not shown; see also Gauthier *et al.,* 2006). Five known or suspected *Drosophila* neuropeptides were not analyzed in these studies for lack of suitable markers-the *Drosophila* immune inducible genes 2 and 4 [18], and neuropeptide-like precursors 2, 3 and 4 [19]. Therefore the genes we could investigate cover the vast majority, but not all of the known neuropeptide genes in the fly. In the following sections, we describe the peptide identities of DIMM-positive cells according to their regional positions within the CNS.

### Identities of DIMM neurons in the 100 hr AEL ventral nerve cord

#### Suboesophageal segments S1–S3

**Figure 5:**
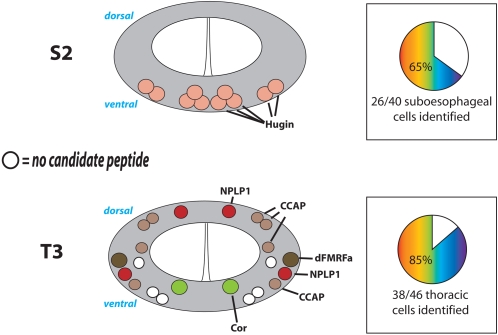
Neuropeptide identities of DIMM cells in the SEG and Thoracic regions of the larval CNS. Neuromeres S2 and T3 are shown as representative of the two regions. The adjoining pie charts indicate the percentages of DIMM-positive neurons in all SEG and thoracic segments respectively that were associated with a specific peptide marker.

There are 40 DIMM-positive cells in these neuromeres and all are found in the ventral aspect: We found three neuropeptide markers expressed among different members of these three neuronal sets, and could therefore identify 65% (26/ 40) of the suboesophageal segment DIMM cells with at least one peptide marker (**Figure 5**). All DIMM-positive neurons in S1 and S2 are Hugin-YFP-positive. Of the 18 DIMM-positive neurons in S3, two are dromyosuppressin- (DMS-) positive, two are CAPA-positive and two are dFMRFa-positive**.** We suspect that the latter two pairs represent the same pair of neurons.

#### Thoracic segments T1–T3

There are 46 DIMM-positive cells in segments T1–T3 and these are found medially and laterally, in both ventral and dorsal aspects. Thoracic DIMM neurons variously express five different peptide markers (for neuropeptide-like precursor 1 (NPLP1), dFMRFa, crustacean cardioactive peptide (CCAP), allatostatin B (Ast-B) and corazonin (COR) (**Figure 5**). We could identify 82% (38/ 46) of the thoracic segment DIMM cells with at least one peptide marker.

#### Abdominal segments A1–A9

**Figure 6:**
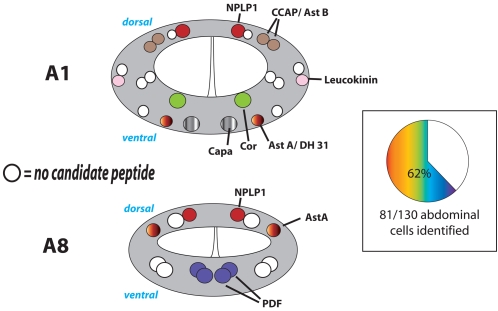
Neuropeptide identities of DIMM cells in the abdominal region of the larval CNS. Neuromeres A1 and A8 are shown as representative of the region. The adjoining pie chart indicates the percentage of DIMM-positive neurons in all abdominal segments that was associated with a specific peptide marker.

Abdominal segments contained between eight (A7) to twenty four (A1) DIMM-positive cells, for a total of 130 DIMM-positive cells in segments A1–A9. Several segmental homologues are present in multiple abdominal segments, including DIMM neurons expressing COR, allatostatin A (Ast-A), ion transport polypeptide (ITP), Diuretic Hormone 31 (DH 31), NPLP1, and leukokinin (LK) (**Figure 6**). In some instances, single DIMM neurons were labeled by multiple neuropeptide markers–antibodies to DH 31 and to ITP and to Ast-A often labeled the same neurons (**Figure 6**). In all, we could identify 65% (85/ 130) of the abdominal segment DIMM cells with at least one peptide marker.

### Identities of DIMM neurons in the brain

**Figure 7:**
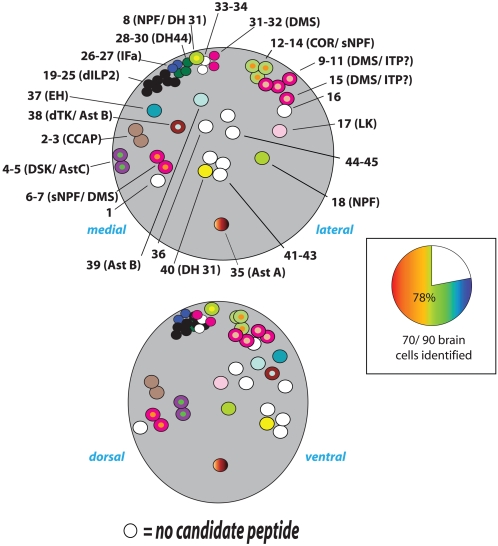
Neuropeptide identities of DIMM cells in the brain. Top diagram illustrates the positions of cells along the medial-to-lateral axis; bottom diagram indicates the positions of cells along the dorsal-to-ventral axis. Cells are numbered arbitrarily, as listed in Table 1. The adjoining pie chart indicates the percentage of DIMM-positive neurons that was associated with a specific peptide marker.

**Figure 8:**
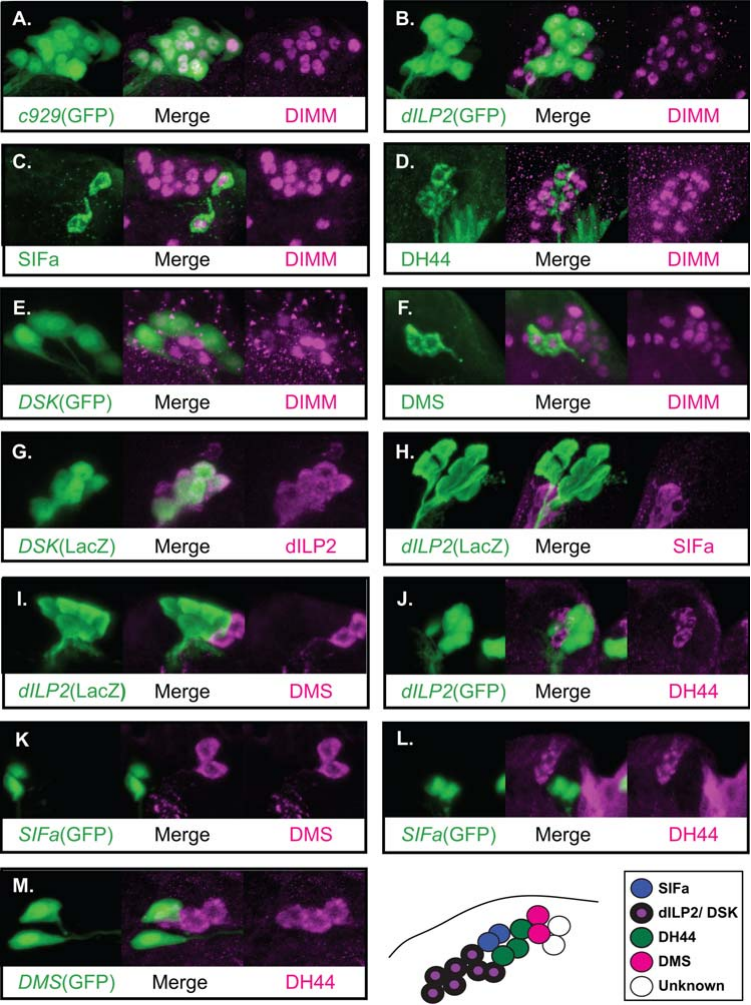
The expression patterns of five neuropeptide markers within DIMM-expressing neurons of the PI. (A) DIMM and *c929*-GAL4 are co-localized within 16 PI neurons. (B) All dILP2-expressing neurons are DIMM-positive. (C) The two SIFa-positive neurons are both DIMM-positive. (D) The three DH 44-expressing neurons are all DIMM-positive. (E) All seven *DSK*-GAL4-positive neurons are DIMM-positive. (F) The DMS-positive neurons are DIMM-positive. (G) The seven *DSK*-GAL4-positive neurons (as visualized with UAS-lacZ) are dILP2-positive. (H) dILP2-positive neurons and SIFa-positive neurons are distinct. (I) dILP2-positive neurons and DMS-positive neurons are distinct. (J) dILP2-positive neurons and DH 44-positive neurons are distinct. (K) The SIFa-positive and DMS-positive neurons are distinct. (L) The SIFa-positive and DH 44-positive neurons are distinct. The results are illustrated in the schematic at the bottom of the figure and in Table 2.

We found that 78% (35 of the 45) DIMM cells per brain lobe could be identified by at least one of the 24 different neuropeptide markers **(** **Table 2** **;** **Figure 7**); in all, seventeen different neuropeptide markers identified diverse, DIMM-positive brain cells. In some instances, single DIMM neurons were labeled by multiple neuropeptide markers. For example, MP1 cells (#4 and 5) expressed drosulfakinin- (*DSK*-)GFP and allatostatin C (Ast-C) immunosignals; the seven *Drosophila* insulin-like peptide- (dILP-) expressing cells (#3-9) were also DSK-GFP-positive; the single DTK cell (#37) was allatostatin B (Ast-B-GFP-)-positive. The Pars Intercerebralis (PI) and Pars Lateralis (PL) are the principle neurosecretory centers of the insect brain (Hartenstein, 2006). Sixteen PI neurons are DIMM-positive (#19-34) and also *c929*-positive (**Figure 8A**). Among the 16 DIMM-positive cells, seven were *dILP2*-GAL4-positive cells, between two and seven were *DSK*-GAL4-positive, three were positive for DH 44, two for SIFa and two for DMS antibodies (**Figure 8B–F**). We asked whether these markers are co-expressed among the PI neurons. All *DSK*-GAL4-positive neurons are also dILP2-positive, while the other neuropeptide markers highlighted unique PI subsets (**Figure 8G–M**). Thus, we found that many *Drosophila* PI neurons express DIMM and at least one of five different neuropeptide markers, dILP2, *DSK*-GAL4, DH 44, SIFa or DMS (**Table 4**).

**Table 2:** Antibodies used for these experiments.

Neuropeptide antibodies	Donor	Dilution	References
Rb anti-AKH (*D. melanogaster*)	S. Kim, Stanford	1∶300	Kim and Rulifson [62]
Rb anti-leucokinin (*L. maderae*	J.Veenstra, Bordeaux	1∶500	Chen *et al*. [82]
Rb anti-FMRFamide (*A. californica*	P. Taghert, St. Louis	1∶2500	Taghert and Schneider [83]
Rb anti-pro-dFMRFa (*D. melanogaster*	R. Scheller, Palo Alto	1∶2000	Chin *et al.* [84]
Rb anti-eclosion hormone (*M. sexta*	J. Truman, Seattle	1∶500	Truman and Copenhaver, [85]
Rb anti-allatostatin C (*M. sexta*	S. Tobe, Toronto	1∶500	Zitnan *et al.* [86]
M anti-allatostatin A (*D. punctata*	B. Stay, Iowa City	1∶2	Stay *et al.* [87]
Rb anti-CCAP (*D. melanogaster*	J. Ewer, Valparaiso	1∶500	Truman and Ewer [88]
Rb anti-proctolin (*D. melanogaster*	D. Nassel, Stockholm	1∶1000	Taylor *et al.* [89]
Rb anti-LMS (*L. maderae*	L. Schoofs, Leuven	1∶500	Schoofs *et al.* [90]
Rb anti-dILP2 (*D. melanogaster*	E. Rulifson, Philadelphia	1∶500	Rulifson *et al.* [91]
Rb anti-corazonin (*D. melanogaster*	L. Roller, Bratislava	1∶1000	Roller *et al*.[92]
Rb anti-s-NPF (*D. melanogaster*	K. Yu, Daejon	1∶250	Lee *et al*.[93]
Rb anti-NPF (*D. melanogaster*	P. Shen, Athens	1∶1000	Wu *et al.* [94]
Rb anti-β PDH (*U. pugilator*	R. Rao, Pensacola	1∶10,000	Nassel *et al.* [95]
Rb anti-DH44 (*D. melanogaster*	J.Veenstra, Bordeaux	1∶500	Cabrero *et al*.[96]
Rb anti-SIFa (*D. melanogaster*	J.Veenstra, Bordeaux	1∶500	Terhzaz *et al*.[97]
Rb anti-IPNa (*D. melanogaster*	L. Schoofs, Leuven	1∶1000	Verleyen *et al*.[98]
Rb anti-pro-CAPA (*D. melanogaster*	J.Veenstra, Bordeaux	1∶1000	Kean *et al*.[99]
Rb anti-DH 31 (*D. melanogaster*	J.Veenstra, Bordeaux	1∶1000	this study
Rb anti-Ast B (*D. melanogaster*	J.Veenstra, Bordeaux	1∶1000	this study
Rb anti-ITP (*S. gregaria*	H. Dircsken, Stockholm	1∶2000	Macins *et al*.[100]

**Table 3:** Transgenic flies used for these studies

GAL4 lines	Donor	References
*CCAP*-gal4	J. Park, Knoxville	Park *et al.* [101]
*CRZ*-gal4	J. Park, Knoxville	Choi *et al*.[102]
*DSK*-gal4	J. Park, Knoxville	this study
*SIFa*-gal4	J.Veenstra, Bordeaux	Terhzaz *et al.* [97]
*DMS*-gal4	J.Veenstra, Bordeaux	this study
*NPF*-gal4	P. Shen, Athens	Wu *et al.* [94]
*dILP2*-gal4	E. Rulifson, Philadelphia	Rulifson *et al.* [91]
*36Y*-gal4	K. Kaiser, Glasgow	O'Brien and Taghert [64]
*Hugin*-YFP, *Hugin*-Gal4	M. Pankratz, Karlsruhe	Melcher and Pankratz [67]
*Mai301*-gal4	Gunter Korge, Berlin	Siegmund and Korge [60]
*Kurs6*-gal4	Gunter Korge, Berlin	Siegmund and Korge [60]
*Feb191*-gal4	Gunter Korge, Berlin	Siegmund and Korge [60]

**Table 4:** Co-expression of peptide markers among the 16 DIMM-positive neurons of the *Pars Intercerebralis*.

	DIMM	dILP2	*DSK*-Gal4	SIFa	DH 44
**dILP2**	Yes	-	-	-	-
***DSK-*** **Gal4**	Yes	Yes	-	-	-
**SIFa**	Yes	No	No	-	-
**DH 44**	Yes	No	No	No	-
**DMS**	Yes	No	No	No	No

In summary, our double staining experiments revealed that 306 of the ∼10,000 neurons of the larval CNS express DIMM protein, while the suite of 23 neuropeptide markers identified ∼1030 diverse neurons. Disregarding known instances of dual neuropeptide expression by single cells, we consider the peptide markers to reveal 530 distinct cells. The rate of false positive discovery of a single peptide marker within DIMM-expressing cells should be roughly [(306/10,000) * (1030/10,000)]*10,000  =  ∼31 cells. More than 200 of the 306 DIMM cells were associated with a specific peptide marker, thus we consider the population of DIMM cells to be highly enriched for a peptidergic phenotype.

### Overlap of DIMM within individual neuropeptide expression patterns

**Figure 9:**
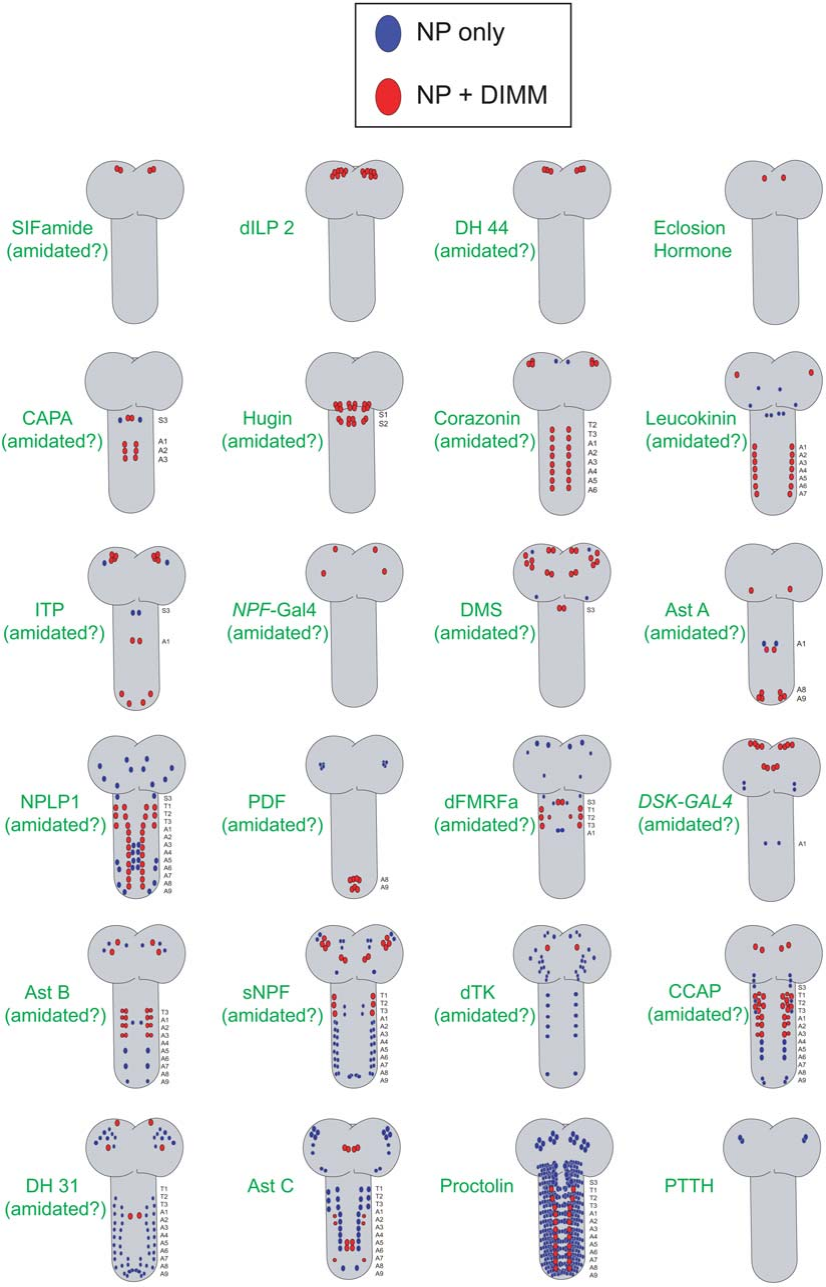
Schematics describing the distribution of cells scored positive for each of the 24 peptide markers found in the 100 hr AEL larval CNS. If the peptides are potentially amidated, this is noted below each marker name in parentheses (amidated). Blue cells are marked by the peptide marker, but lack DIMM staining; Red cells co-express DIMM. The proctolin schematic does not show the complete complement of ∼400 proctolin-immunoreactive neurons. Schematics are distributed with ones showing greater percentages of DIMM co-expression are towards to the top of the figure and ones showing lesser percentages are towards the bottom.

**Figure 10:**
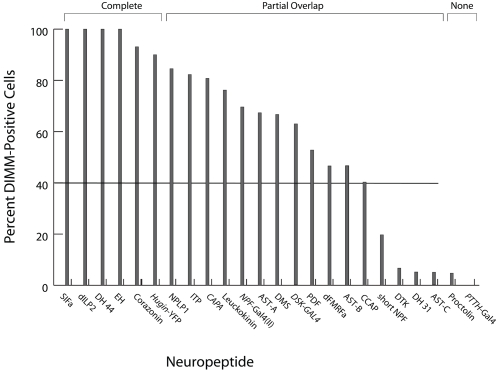
The quantitative representation of DIMM co-expression among sets of cells expressing any of 22 different peptide markers in the 100 hr AEL larval CNS. 16 of 22 peptide markers display DIMM co-expression in more than 40% of the peptide-expressing cells (horizontal bar). The degree of overlap is categorized as “Complete”, “Partial”, or “None”, as described in the text.

**Figure 9** presents schematic diagrams to illustrate the overlap of DIMM immunosignals with 24 different neuropeptide markers in the larval CNS. **Figure 10** quantifies the same data. We observed three patterns of co-localization of DIMM antibody signals with those for individual neuropeptide markers. (i) Complete Overlap was displayed by 6/24 markers: >90 % of peptide-expressing cells were also DIMM-positive. (ii) Partial Overlap was displayed by 16/24 markers: between 4 and 90% of peptide-expressing cells were also DIMM-positive. (iii) Virtually No Overlap was displayed by 2/24 markers: <4% of peptide-expressing cells were also DIMM-positive. Examples of the co-localization between DIMM and neuropeptide markers for each of the three categories are shown in **Figure 4**. Here we describe each category in turn.

#### Category A: Complete Overlap with DIMM

*Hugin*-YFP (marks 22 neurons located in subesophageal segments S1 and S2 [49]): 20 of these are strongly DIMM-positive (**Figure 4A**). Likewise SIFa- (**Figure 8C**), eclosion hormone- (EH-), dILP2- (**Figure 8B**), Diuretic hormone 44- (DH 44-) (**Figure 8D**), and COR-expression markers (**Figure 4B**) all displayed greater than 90% overlap with DIMM.

#### Category B: Partial Overlap with DIMM

The extent of partial overlap was very broad among different peptide markers, but reproducible for individual markers. For example, eight of twelve allatostatin A (Ast-A-)-positive cells were DIMM-positive (**Figure 4C**), and most CAPA-expressing neurons were all strongly DIMM-positive. In the case of CCAP, we saw that 12 of the 29 peptidergic cells were DIMM-positive (**Figure 4D**). There were 32 dTK-positive cells but only two were DIMM-positive (**Figure 4E**). Other peptidergic systems displaying partial overlap included DSK (**Figure 8E**), DMS (**Figure 8F**), Ast B, Ast C, LK, neuropeptide F (*NPF*-GAL4), pigment dispersing factor (PDF), dFMRFa, ITP, short neuropeptide F (sNPF), NPLP1, and DH 31.

To assess how reproducible are partially overlapping patterns we counted the incidence of peptide marker and DIMM overlap in a large cohort of specimens for each of two peptides in this category– dTK and LK. For DTK, we counted an average of 29.7 +/− 0.57 peptidergic neurons (n = 21 specimens and each had exactly 2 neurons that were DIMM-positive. For the case of LK, we counted an average of 23.17 +/− 0.43 peptidergic neurons (n = 17 specimens), of which 17.65 +/− 0.28 were DIMM-positive. In both dTK and LK systems, the DIMM-positive neurons appeared to be a reproducible subset, as judged by cell body position.

An additional feature that described the ‘Partial Overlap’ category was the strong correlation between DIMM-co-expression and intensity of peptide marker expression. For example, among CCAP-expressing neurons, strongly-stained neurons were invariably DIMM-positive and “less-strongly” stained ones were DIMM-negative (**Figure 4C**). This correlation was also clearly evident in the co-expression patterns for PDF, COR, DMS, dFMRFa, LK, dTK, NPLP1, CAPA-, ITP-, Ast A and Ast B. The example of PDF:DIMM coincidence is especially interesting as the DIMM-negative LNv (see also Taghert *et al.,* 2001) are implicated in control of circadian locomotor rhythms via neuropeptide PDF release (recently reviewed by [50]). The sNPF, DH 31 and Ast C patterns present three prominent exceptions to that general rule. Specifically, the most strongly-staining cells in these groups were reproducibly not DIMM-positive. sNPF and DH 31 peptides are potentially amidated; the AstC peptide is not. Thus sNPF and DH31 systems were exceptions to this general rule. These systems can produce amidated peptides according to their genomic sequences and amidated forms have been recovered by purification or peptidomic analyses[51].

#### Category C: Virtually No Overlap with DIMM

Two peptide markers displayed little if any overlap with DIMM. The two prothoracicotropic hormone (PTTH-) producing neurons of the brain did not express DIMM and the widely-expressed pentapeptide proctolin (>400 proctolin-positive neurons per larval CNS) overlapped with DIMM only in the 24 Ap-let neurons (**Figure 4F**). Ap-let neurons are peptidergic [35] and recently were shown to express the NPLP1 neuropeptide ([52] and **Figure 9**). They were weakly proctolin-immunoreactive.

### Relationship between neurons expressing DIMMED and Ddc

Co-expression of peptide and bioactive amine transmitters is a common observation in many different model systems [53]. We wondered whether DIMM cells also co-express small conventional transmitters. Using *c929*-GAL4 (the *dimmed* reporter) and a specific anti-Ddc [54], we previously reported a strict segregation of *c929* and Ddc immunosignals in the larval CNS[33]. We re-examined this question with a *Ddc*-GAL4 line and DIMM antbody staining. Surprisingly, we found that the prominent CRZ-positive neurons in the ventral aspect of segments A2-A7 were *Ddc*:GAL4-positive, as were the PDF-expressing mid-line neurons of segments A8 and A9, and the dorso-lateral CCAP-expressing neurons of segments A1-A4 (data not shown). All these cells were DIMM antibody-positive (**Figure 6**). We consider possible explanations for this different result in the Discussion.

## Discussion

To evaluate and interpret the phenotypic traits associated with normal expression of *dimmed*, we have employed three independent anatomical measurements–(i) mRNA *in situ's,* (ii) regulatory promoter sequences [33], [34], and (iii) specific antibody staining ([40], and this study). Expression starts in mid-embryonic stages in 200-300 cells of the CNS and in several endocrine cells. In most of these cells, *dimm* expression carries forward stably through all subsequent developmental stages. The *dimm in situ* pattern was highly reminiscent of the *c929*-GAL4 pattern [33]. Here we have shown that the *c929*:GAL4 pattern is precisely matched by the expression of DIMM immunosignals (**Figure 1**). These signals are essentially lost in *dimm* mutant backgrounds ([33]; this study, **Figure 1**) validating the interpretation that DIMM is normally restricted to a widely-distributed, but numerically-restricted subset of central and peripheral cells. Therefore, the map of DIMM-expressing cells we present is based on a foundation of independent methods, which together produce highly congruent results. That internal consistency increases the value of our subsequent efforts to identify and characterize DIMM-expressing cells in the aggregate, and as individual cells.

### Physical mapping of DIMM cell bodies

We used the larval brain mapping atlas of Perneau and Hartenstein [48] to fix approximate locations to the DIMM-expressing cells in the hemispheres. We found that the ∼45 DIMM cells in each hemisphere occupy reproducible positions in proximity to one or more identified secondary lineage. Secondary lineages are defined as the clones derived from post-embryonic divisions of the ∼100 neuroblasts per brain hemisphere [48], [55]. The lineage histories of insect peptidergic neurons are largely unknown, but the physical association of DIMM neurons with specific secondary lineages suggests the potential for assigning clonal relationships to them. The lineage history of DIMM cells as a group is of fundamental significance because these cells comprise a large fraction of the most significant peptidergic neurosecretory neurons of the *Drosophila* brain. The cell lineage of certain of the CRZ-, dFMRFa- and NPLP1-expressing DIMM neurons have been described [52], [56], [57]. The distributed and largely invariant positions of DIMM cells in the CNS suggest their derivation from numerous, different NBs, but this supposition awaits future experimental analysis. 16 of the 45 DIMM cells in the brain are likely not derived from specific neuroblasts as they are found within the PI region of the protocerebrum. The PI is one of the major insect brain neuroendocrine centers [58] and its developmental origins from ectodermal placodes have recently been described by de Valasco *et al.* [59]. Siegmund and Korge [60] used random GAL4-generated reporter activity to identify as many as 18 PI neurons that innervate the Ring Gland in each *Drosophila* brain hemisphere. We found that all of the PI neurons revealed by the *Jan191*-, *Mai301*- and *Kurs6*-GAL4 lines were DIMM-positive (unpublished observations).

### Most or all DIMM cells are peptidergic

DIMM was originally described in the context of peptidergic neuronal expression [33], but the possible restriction to that cellular class was never quantified. The present results confirm that many DIMM cells are in fact peptidergic and suggest that most may be. Using markers for ∼24 peptide-encoding genes, we could assign peptide identities to 64% of the 306 DIMM-expressing cells in the 100 hr AEL larval stage *Drosophila*. Furthermore, the broad representation of DIMM among most of peptide markers here surveyed (only two or three of 24 markers lacked substantial DIMM expression) suggests the percentage of identifiable DIMM cells will increase as markers for other *Drosophila* peptide systems become available. We used specific anti-peptide antibodies and neuropeptide GAL4 lines where available (**Tables 2** **&** **3**) and refer to these as “peptide markers” because these have not all been verified to be 100% authentic expression patterns. For example, there could be cross-reactivity between markers (especially significant for markers of the various RFamide-and PRXamide-containing peptides) but that would not preclude their inclusion in this effort to map the potential peptidergic character of DIMM-positive cells. With respect to the incidence of co-transmitter expression among peptidergic DIMM-positive neurons, we found that a few DIMM antibody-positive in the VNC were *Ddc*-GAL-positive neurons. The *Ddc*-GAL4 pattern appears exhuberant compared to that demonstrated by the anti-Ddc antibody ([33], [54] and B. White, pers. communication). One possible conclusion is that, unlike other peptidergic neurons. DIMM cells are dedicated to a very high-level peptidergic function (see below) and so cannot also sustain expression of conventional co-transmitters.

### Where DIMM fits in among peptidergic cells

**Figure 11:**
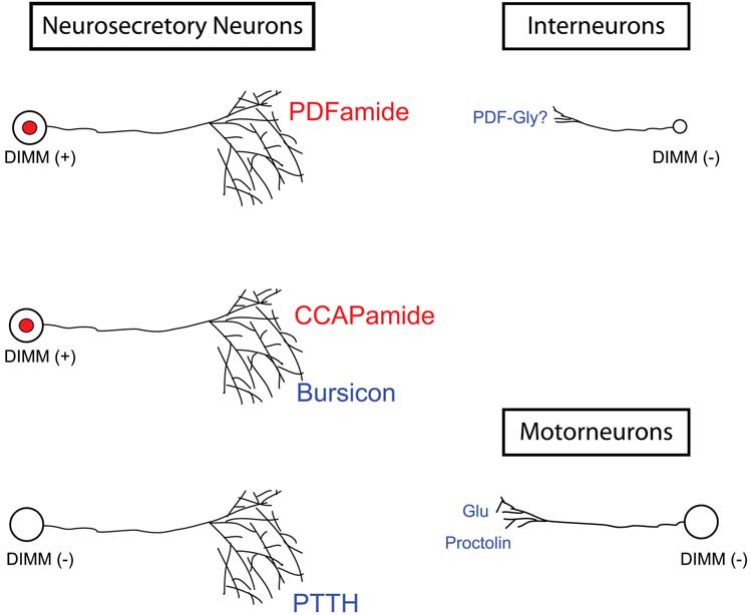
Classification of peptidergic neurons in *Drosophila* according to several physical and biochemical attributes based on the observations reported in this manuscript. We propose that DIMM neurons are peptidergic Neurosecretory Neurons whose distinctive properties are enumerated by the acronym LEAP. They are distinguished by their Large size (long axon morphology and extensive arborizations), periods of Episodic release high levels of secretory activity and by specific modifications of secretory products (Amidation of Peptides). The top row compares two peptidergic neuronal classes in the *Drosophila* CNS, using the example of neuropeptide PDF expression. The DIMM-positive Neurosecretory Neuron produces high levels of amidated PDF. The DIMM-negative Interneuron produces more modest levels of amidated PDF, or more likely of Gly-extended (non-amidated) PDF. DIMM neurons express peptides that can be amidated. They may also express peptides or protein hormones that are not amidated, as illustrated for the example of Neurosecretory Neurons that express both CCAP (potentially amidated) and Bursicon (a non-amidated, glycoprotein hormone). Neurosecretory Neurons that lack DIMM express only non-amidated secretory products (bottom left). Motorneurons that co-release glutamate (Glu) with the non-amidated peptide Proctolin (bottom right) also lack DIMM. See text for further details.

Peptidergic cells are not easily classified–they may be large or small, express different neuropeptides, may have varying levels of peptide output, and they may modify their secretory peptides post-translationally in several alternative ways. To discuss the possible roles of DIMM in peptidergic cell biology of *Drosophila*, we illustrate a range of cellular phenotypes in **Figure 11**. This range distinguishes neurons according to three main attributes–(i) physical size, (ii) physiological status (meaning, the level of secretory activity or suspected cellular class) and (iii) biochemical activity (specifically, post-translational modifications of the secreted peptides). DIMM distinguishes a precise subset of peptidergic cells, but not according to peptide identity. Rather, we propose that DIMM is normally associated with those peptidergic cells that (i) are large cells and not small, (ii) are neurosecretory cells and not interneurons or motorneurons (i.e., highly active in peptide production and episodic release), and that (iii) amidate their secretory peptides post-translationally. To symbolize the amalgamation of these three properties into a singular, genetically-defined cell fate we propose calling DIMM-expressing cells LEAP cells–Large cells that Episodically release Amidated Peptides. Next, we discuss each of these three ideas separately.

### DIMM peptidergic cells are relatively large

We found DIMM as a normal component of many of the classic and well-studied *Drosophila* neuroendocrine systems–these include AKH-expressing cells of the corpora cardiaca [61]–[63], ETH-expressing cells of the Inka system [64], [65], a large fraction of the classic Pars Intercerebralis[66], the hugin-expressing neurons of the subesophageal segments [67] and the Tv neurons of the thoracic segments [68]. These DIMM-positive neural and endocrine cells must attain large cell body size in part to sustain production of hormone levels sufficient to release effective doses into the general circulation. Beyond the association with classic neurosecretory systems, we noted that DIMMED expression is also highly predictive of greater size among cells co-expressing a single peptide marker. For example, in the larval CNS there are two sets of neurons expressing the neuropeptide PDF (**Figure 11**): (i) the eight LNv's of the brain are interneurons and DIMM-negative (they later become critical circadian pacemakers in the adult CNS–[69]), and (ii) a group of 4–6 neurons of abdominal segments A8 and A9 that project long axons onto the hindgut. This second PDF-expressing group is Neurosecretory (neuroendocrine) and DIMM-positive; these cells have large cell bodies and considerable amount of axonal arbor (**Figure 11**, left side). Thus DIMM neurons display large cell bodies, are more likely to extend a peripheral axon, or display an axon that spans the entire length of the CNS. This interpretation is partly circular: estimations of cell body size and visibility of neuronal arbor are highly dependant on the intensity of anti-peptide antibody staining, which we have shown to be critically dependent on DIMM levels [33], [34], [40]. Nevertheless, in considering the physiological contributions that DIMM may make to the differentiation and organization of peptidergic cells, we speculate that the issue of size is highly pertinent to the biology of DIMM neurons.

To further emphasize the correlation of normal DIMM expression and cell body size, we note that in the adult CNS, the small LNv remain DIMM-negative, while the newly-differentiated large LNv are DIMM-positive ([70] and data not shown). This positive correlation between DIMM expression and increased cell size also holds for other markers in the “**Partial Overlap**” category, including: dTK, dFMRFa, DSK, DMS, NPLP1, NPF, Ast-A, and LK cells. Therefore, we propose DIMM neurons are large, they often projecting axons to the periphery or to long distances within the CNS. They are distinguished by these features from most other peptidergic interneurons (like the PDF-expressing interneuronal LNv) which are more diminutive.

### DIMM peptidergic cells are neurosecretory and display a high physiological state

The classic features of neurosecretory function are the production and release of large amounts of hormone(s): the Bag Cells of *Aplysia* dedicate 50–70% of protein synthesis to production of the peptide Egg Laying Hormone [71]. Likewise, individual vasopressin-secreting magnocellular neurons are estimated to contain 2,000 molecules of vasopressin mRNA per cell, and oxytocin-secreting neurons contain 5,000 to 12,000 molecules of oxytocin mRNAs per neurosecretory cell [72]. As mentioned above, DIMM is a normal molecular constituent of most of the classic peptide neurosecretory systems of *Drosophila*. Therefore, we propose that DIMM cells display, on average, a more highly-active secretory profile that conventional interneurons or motorneurons–and that DIMM is therefore essential to define a prevalent class of Neurosecretory Neuron in the fly (**Figure 11**, top left). This class comprises neuroendocrine cells that project into the periphery to form neurohaemal endings and release products into the circulation. Likewise we propose the DIMM Neurosecretory class also includes the large DIMM-positive peptidergic neurons that extend axons long distances and maintain large axonal arbors, but which remain within the CNS. Examples of that category include the DIMM-positive dTK neuron (#38, **Table 1**), the NPF-positive cell (#8–**Table 1**) and the sNPF/DMS- positive cells (#4-5, **Table 1**).

We further speculate that DIMM cells are defined by the common physiological property of displaying Episodic Release. The term refers to brief periods of intense secretory activity that delivers large amounts of stored peptide quickly. DIMM cells already known to display this property include the Inka cells [73], EH cells [7], and Bursicon/CCAP cells [11]. By analogy with studies in other insects [74], we presume that the DIMM-positive *Drosophila* AKH cells also release episodically. By analogy with the rapid post-prandial activation of mammalian insulin-producing cells (reviewed by [75]), we presume dILP-2-producing neurons of the *Drosophila* PI also display episodic release. Additional examples in *Drosophila* derive from recent physiological studies of ETH actions. The peptide hormone ETH triggers sequential (episodic) waves of activation in diverse peptidergic target neurons in the larval CNS [76]. The first cells to respond are the DIMM-positive Tv neurons (expressing dFMRFa), followed by the EH cells, then DIMM-positive CCAP cells (cell 27/703), and finally the DIMM-positive Bursicon/CCAP-containing of the abdominal segments. Hence for many of the DIMM neurons about which we have at least some information concerning their activity, they undergo release events episodically, as indicated by the sudden, temporally-restricted manner in which they are activated.

High-levels of steady-state peptide antibody staining may be explained by affecting any of three cellular properties: (i) increased peptide synthesis/ accumulation, (ii) decreased peptide release or (iii) decreased peptide turnover. Most of the available evidence concerning DIMM functions cannot distinguish between these potential explanations, and each may have validity. However, it now well established that DIMM is a strong activator of the neuropeptide biosynthetic enzyme *PHM* [34], [40] and this means that DIMM is a pro-secretory regulatory factor. This is evidence to support the first explanation and hence we interpret DIMM-dependent changes as not simply the prevention of neuropeptide release or turnover. We favor the interpretation that the individual DIMM cells are the most strongly-stained for secretory peptide products because they display the highest levels of secretory activity.

Our speculations are based on correlating the cellular properties of neurons that normally produce DIMM, as shown in this report. In addition, they are supported by several, previous experimental studies: genetic analyses employing *dimm* loss- and gain-of-function states [33], [34], [40], [43]. In particular, the *dimmed* loss of function phenotype reflects a decline in steady-state levels of peptides and peptide biosynthetic enzymes [33]. DIMM does not influence cell survival, and the mutant neurons retain the ability to produce at least a certain low-level of peptidergic production. Thus *dimm* is not required to initiate the differentiation of a peptidergic phenotype but instead appears necessary for the full, quantitative display of the peptidergic neurosecretory phenotype. In similar fashion, the mammalian sequence orthologue of *dimm*, called *Mist1*, is not needed for survival or initial specification of the Chief cells as secretory cells of the stomach. Instead it is needed for their complete differentiation as zymogenic cells, which normally display a robust secretory phenotype [39].

### DIMM peptidergic cells specifically express amidated peptides

The third distinguishing feature we highlight is the strong correlation of DIMM with C-terminal peptide amidation. Neuropeptide amidation is a post-translational modification of many neuropeptides–about 50% of peptides in mammals [77] and greater than 90% of peptides in *Drosophila* are amidated [14]. Amidation can affect the turnover rate of secreted peptides and/ or affect the binding or activation of receptors by secreted peptides (reviewed by [77]). DIMM directly regulates the *PHM* gene which encodes the rate-limiting enzyme for peptide amidation [40], and its expression is highly congruent with that of PHM[33], [34].

If this regulatory connection is meaningful, then normal DIMM expression should be highly correlated with that of amidated peptides. In addition, it should not show any particular correlation with expression of non-amidated peptides. We note that very few anti-peptide antibodies are known to distinguish between amidated and Gly-extended forms of their peptide antigens. Thus, “actual” amidation states of peptides which may be amidated is uncertain for any given neuron. However, peptide amidation displays specific sequence requirements and neuropeptides that do not show these (e.g., Ast-C, proctolin and certain large protein hormones) do not display that modification. Given these considerations, how well were these predictions met? At first glance, the results were seemingly mixed, however closer inspection reveals that fundamentally the predictions hold true. On the one hand, DIMM is widely expressed by numerous cells expressing diverse, amidated (amidatable) peptides and this result conforms to the prediction. Counter to the hypothesis however, we found that DIMM is also reproducibly expressed by several classic neurosecretory, peptidergic neurons that express large, non-amidated peptide hormones–the fourteen dILP-neurons, the two Eclosion Hormone neurons [78] and in the several bursicon neurons [79]. In addition, DIMM is found in certain neurons that express Ast-C and proctolin. These counter-examples suggest the initial hypothesis of equating DIMM with exclusive expression of amidated peptides cannot be supported in its simplest terms.

However, we note that DIMM is found in Ast-C neurons that are only weakly-stained by Ast-C antibodies, not in ones stained more strongly–hence in this case, there is no correlation of DIMM with “strong” expression of a non-amidated peptide, in contrast to the situation for numerous amidated peptides (**Figures 4** **,** **9** **and** **10**). Furthermore, pancreatic β-cells of mammals express insulin, but also co-express the amidated peptide amylin [80]. Therefore, DIMM expression in insect dILP2-, EH-, Bursicon-, in some Ast C- and some proctolin-expressing neurons may be explained by invoking a second class of Neurosecretory neurons in *Drosophila* (**Figure 11**, middle left.): these produce peptides that be amidated along with non-amidated peptides or protein hormones. In fact this appears true for at least some examples here mentioned: bursicon-containing neurons co-express a peptide that can be amidated-CCAP [79]. The DIMM-positive proctolin neurons are called Ap-lets [35] and they express the precursor NPLP1 which also encodes peptides that can be amidated [52]. Likewise, we here show that dILP neurons co-express a genetic marker for *DSK*, which encodes peptides that can be amidated. On this basis, and in support of our initial hypothesis concerning DIMM's role, we predict the EH-secreting neurons normally co-express an amidated peptide. To invoke such an amidated peptide is not unreasonable, as currently we lack markers for several known *Drosophila* amidated peptides, such as for example, the Drosophila immune-inducible peptides 2 and 4 [18].

We found one classic peptidergic Neurosecretory system in *Drosophila* that does not express DIMM–the PTTH-expressing neurons of the brain. These resemble DIMM Neurosecretory Neurons in that they have large cells bodies, extensive axonal projections, and display high levels of steady-state peptide accumulation and episodic release. These cells may be DIMM-negative because they only express DIMM transiently, during a developmental stage that we did not survey. However, we favor the explanation that these neurons possess an alternative but regulatory system: comparable, but distinct from that controlled by DIMM. We note that PTTH is a large glycoprotein hormone that is not amidated and that (at present) we have no evidence for any expression of peptides that can be amidated in these cells. Therefore we predict *Drosophila* contains a third class of Neurosecretory Neuron (**Figure 11** **,** lower left)–a DIMM-negative one that only produces non-amidated secretory products.

Finally, for the class of Neurosecretory Neurons that co-express amidated and non-amidated peptides (**Figure 11**, middle left), we wonder whether DIMM controls the secretory pathway for one or both sets of secretory products. It is interesting to consider for these cases, that amidated and non-amidated secretory peptide pathways within single neurons may be controlled by distinct regulatory cascades. To evaluate this possibility, it will be useful to determine whether manipulation of DIMM levels leads to changes in steady-state levels of the non-amidated secretory peptides and peptide hormones, namely, EH, bursicon, proctolin, Ast C and the dILPs.

## Materials and Methods

### Fly stocks

We used Canton S for a wild type stock; GAL4 transgenic lines as described in **Table 3** were crossed to either of two UAS-reporter stocks (*2X EGPF* or -*lacZ*). Flies were reared and maintained on a standard cornmeal-agar medium at room temperature (22-23^o^C). *DSK*-GAL4 stocks were generated as follows: a 706-bp DSK upstream sequence was PCR-amplified from wild-type genomic DNA template, the 3′ end of which is two base pairs upstream of the translation start site. PCR primers were GCTCTAGA-TGGGTATCGTGTTAATATCAG (forward primer with Xba I site) and GCGGTACC-ACAGCGTGGCGAAGTGCGTA (reverse primer with Kpn I site). PCR product was digested with Xba I and Kpn I, the insert was cloned into *P{pPTGAL}* vector, and resulting construct employed for germ-line transformation. *DMS*-gal4 enhancer flies were produced by amplifying the putative *DMS* promoter using the following primers: GCGAGATCTCGGTGCTTCCACAAAGAAGT and GCGGAATTCCGCAAAGTGGCGAAAATAAT. The PCR product was cloned into the P{*pAKH*-*GAL4*} vector [61] digested with Eco R1 and Bam H1. Transgenic flies were produced by TheBestGene Inc. (www.thebestgene.com).

### Antibodies, Immunostaining and Imaging

Immunostaining methods were as previously described by Hewes *et al*. [33]. The CNS of the 90–100 hr (feeding IIIrd instar) AEL larvae were dissected in the standard saline that lacked calcium and fixed with 4% paraformaldehyde / 7% picric acid (v/v) in 1X PBS. Embryos were harvested from egg collection plates, dechorionated with 50% chlorine and fixed with 37% formaldehyde for 3 min in 50% heptane, then washed with 100% methanol. The primary antibodies used as peptide markers in this study are listed in **Table 2**. Anti-β-galatosidase antibody (Promega, WI, 1∶1000), MAb anti-neurotactin (BP106; Developmental Hybridoma Bank, Iowa City, 1∶100), MAb 4D9 anti-inv (gift from J. Skeath; 1∶10), rabbit anti-EVE (gift from J. Skeath; 1∶500), were also used. Antisera were raised to *Drosophila* DH 31, a kind gift from Julian Dow and to *Drosophila* Ast-B AWQSLQSSWamide (Research Genetics, Huntsville, AL). In both cases the peptides were coupled to porcine thyroglobulin using difluorodinitrobenzene as described by Tager [81], in a ratio of 2 mg peptide to 5 mg carrier protein. Unreacted peptide was removed by dialysis and the conjugate injected in five to six sites on the back of a female New Zealand white rabbit. Booster injections were given at six week intervals. Blood was collected before the first injection and ten days after each booster injection; serum was collected and stored frozen. Cy3-conjugated, Alexa-568, Alexa-633 or Alex-488-conjugated, secondary antibodies were used at 1∶500 dilutions. Images were acquired on an Olympus FV500 laser scanning confocal microscope and manipulated by Adobe Photoshop software to adjust contrast. For the positional analysis of larval brain DIMM cells, the images acquired from the confocal microscope were imported into and analyzed with Amira software, as described at Pereanu and Hartenstein[48].

## Supporting Information

Figure S1 Double antibody staining for c929-GAL4 activity (green) and DIMM immunoreactivity (magenta) in a single confocal image of the 100 hr AEL larval CNS. The single channel for DIMM antibody staining is shown on the right. Strongly-stained cells for each marker were highly correlated (arrows); hands illustrate weakly-stained cells. Only the strongly-stained cells were scored in this report.
(3.62 MB TIF)

Figure S2 Neuropeptide identities of DIMM neuroendocrine cells in the periphery. The sixteen cells of the corpora cardiaca (CC) that express the peptide hormone AKH are all DIMM-positive. The 14 Inka cells associated with the largest tracheal trunks and that express the peptide hormone ETH are all DIMM-positive. Seven neurons in the oesophageal ganglion of the SNS that express unidentified -RFa-positive neurons are DIMM-positive. No specific peptide marker has yet been associated with the 14 DIMM-positive LBD neurons that are situated along the segmentally-repeated Transverse Nerve. In other insects, a similar neuron is peptidergic (Wall and Taghert, 1990). The pie chart indicates the percentage of all DIMM-positive peripheral cells that have been associated with a specific peptide. All DIMM-expressing peripheral cells are PHM-positive (not shown).
(0.33 MB TIF)

Table S1 Quantification of overlap between DIMM immunostained cells and 24 different peptide “markers” (antibodies or GAL4 lines) in the 100 hr AEL larval CNS.
(0.03 MB DOC)
