# Supplementary figures and images for: Mapping Peptidergic Cells in Drosophila: Where DIMM Fits In
Source: PLoS One. 2008 Mar 26;3(3):e1896. doi: 10.1371/journal.pone.0001896 (PMC2266995; doi:10.1371/journal.pone.0001896)

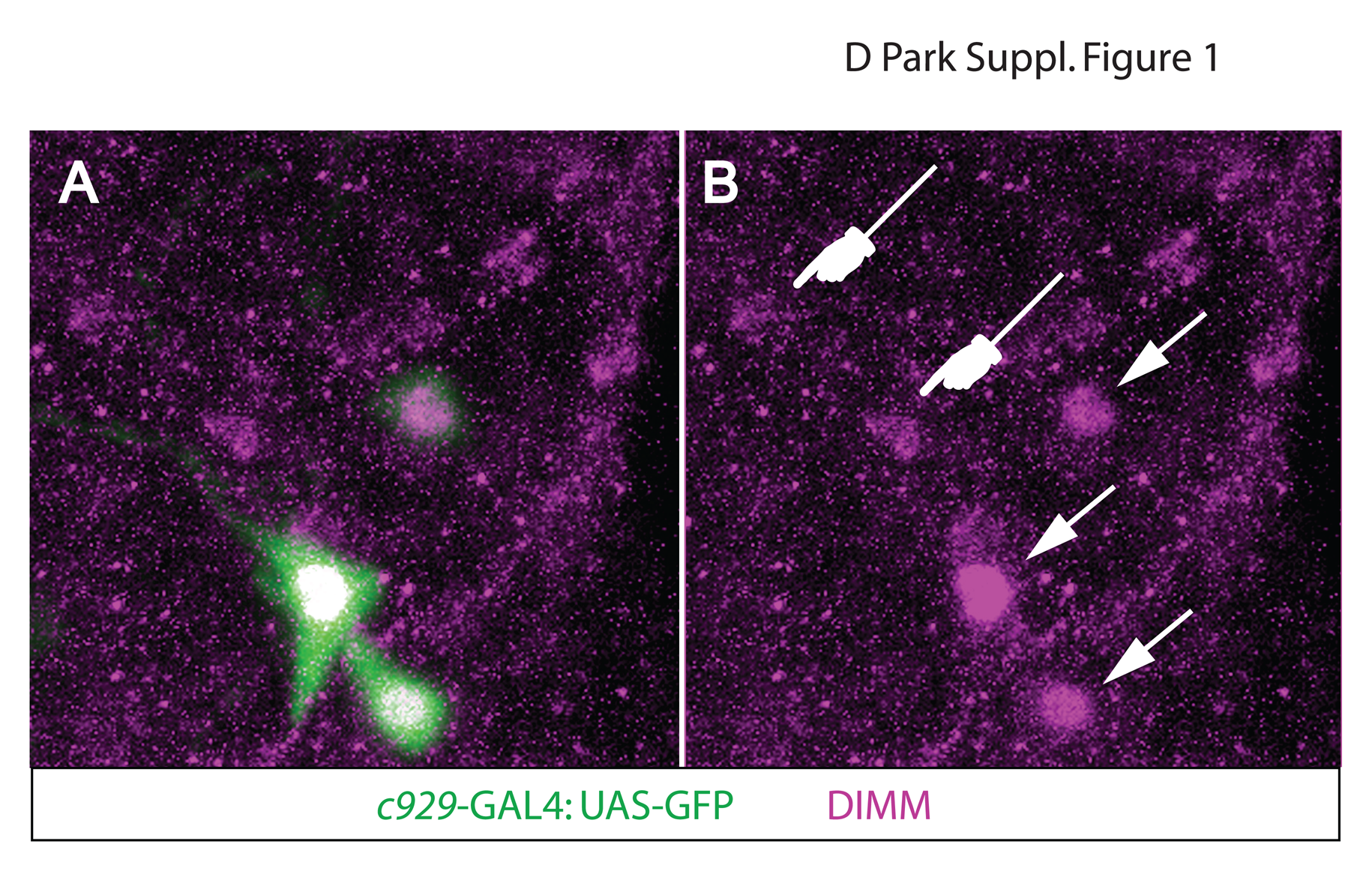

Supplement: Figure S1 — Double antibody staining for c929-GAL4 activity (green) and DIMM immunoreactivity (magenta) in a single confocal image of the 100 hr AEL larval CNS. The single channel for DIMM antibody staining is shown on the right. Strongly-stained cells for each marker were highly correlated (arrows); hands illustrate weakly-stained cells. Only the strongly-stained cells were scored in this report. (3.62 MB TIF) [file pone.0001896.s001.tif]

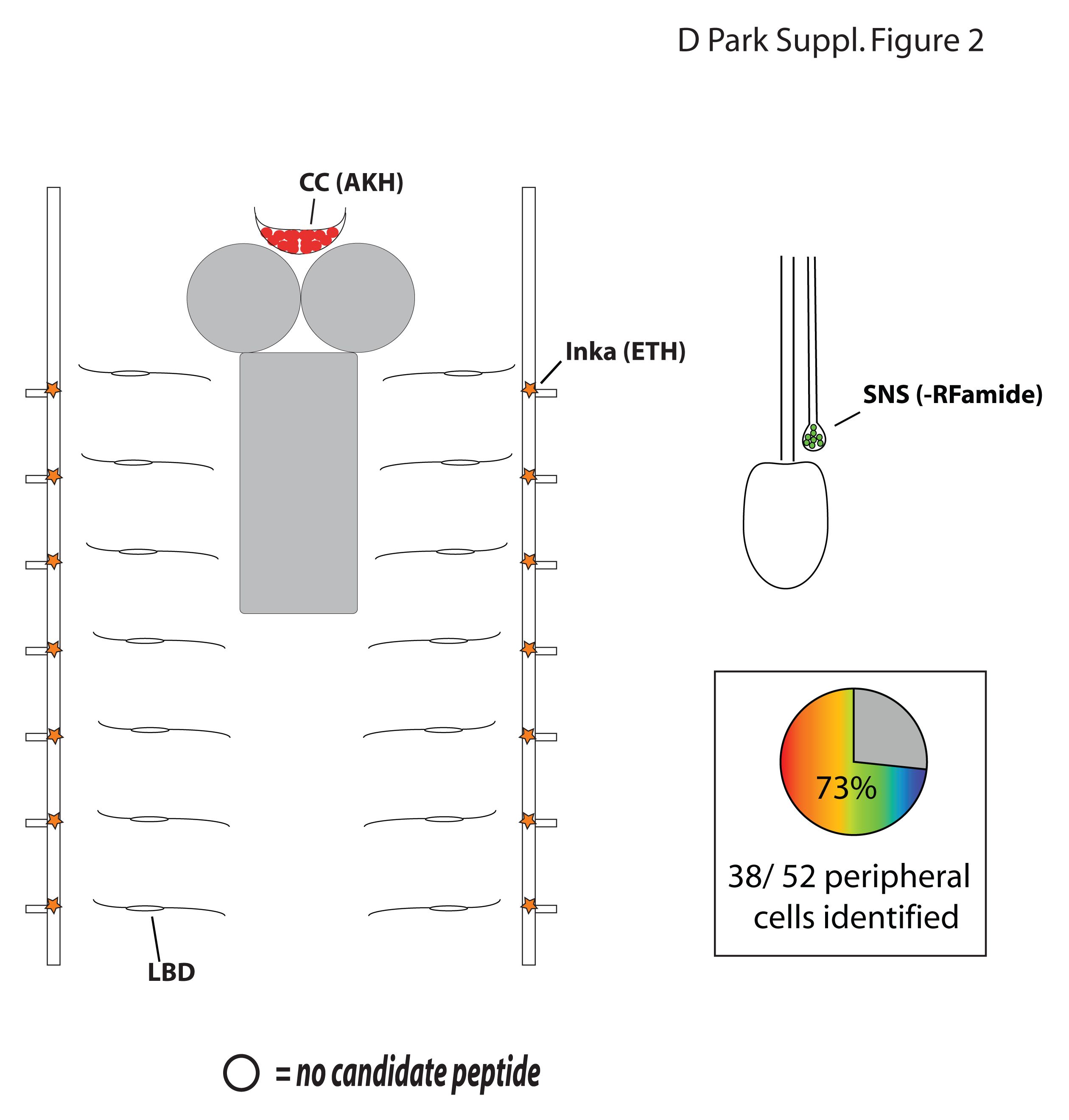

Supplement: Figure S2 — Neuropeptide identities of DIMM neuroendocrine cells in the periphery. The sixteen cells of the corpora cardiaca (CC) that express the peptide hormone AKH are all DIMM-positive. The 14 Inka cells associated with the largest tracheal trunks and that express the peptide hormone ETH are all DIMM-positive. Seven neurons in the oesophageal ganglion of the SNS that express unidentified -RFa-positive neurons are DIMM-positive. No specific peptide marker has yet been associated with the 14 DIMM-positive LBD neurons that are situated along the segmentally-repeated Transverse Nerve. In other insects, a similar neuron is peptidergic (Wall and Taghert, 1990). The pie chart indicates the percentage of all DIMM-positive peripheral cells that have been associated with a specific peptide. All DIMM-expressing peripheral cells are PHM-positive (not shown). (0.33 MB TIF) [file pone.0001896.s002.tif]
