# Supplementary material for: Mapping Peptidergic Cells in Drosophila: Where DIMM Fits In
Source: PLoS One. 2008 Mar 26;3(3):e1896. doi: 10.1371/journal.pone.0001896 (PMC2266995; doi:10.1371/journal.pone.0001896)
Supplement: Table S1 — Quantification of overlap between DIMM immunostained cells and 24 different peptide “markers” (antibodies or GAL4 lines) in the 100 hr AEL larval CNS. (0.03 MB DOC) [file pone.0001896.s003.doc]

# **Table S1.** Quantification of overlap between DIMM immunostained cells and 24 different peptide “markers” (antibodies or GAL4 lines) in the 100 hr AEL larval CNS.

**Neuropeptide Total cell # # DIMM-positive %DIMM-positive # specimens**

## SIFa 3.8 +/- 0.1 3.8 +/- 0.1 100 7

## dILP2 14 +/- 0 14 +/- 0 100 3

DH 44 6 +/- 0 6 +/- 0 100 3

EH 2 +/- 0 2 +/- 0 100 7

Corazonin 23.6 +/- 0.2 22 +/- 0 93.1 8

*Hugin*-YFP 22 +/- 0 20 +/- 0 90.9 4

## NPLP1 33.3 +/- 1.8 27.3 +/- 0.7 82 3

ITP 18.1 +/- 0.6 14.6 +/ - 0.3 80.3 7

CAPA 10 +/- 0 8 +/- 0 80 3

Leuckokinin 23.2 +/- 0.4 17.7+/- 0.3 76.2 17

## *NPF*-Gal4(II) 5.8 +/- 0.3 4 +/- 0 69.6 4

# Ast-A 12 +/- 0 8 +/- 0 66.7 4

# DMS 23.3 +/- 1.2 15.5 +/- 1 66 6

## *DSK*-Gal4 14.9 +/- 1 8.9 +/- 1.9 59.7 9

PDF 16.8 +/- 0.2 8.8 +/- 0.2 52.8 5

dFMRFa 25.8 +/- 0.9 12 +/- 0.4 46.6 4

# Ast-B 21.7 +/- 0.3 10 +/- 0.6 46.2 3

CCAP 28.9 +/- 1.3 11.6 +/- 1.3 40.3 8

short NPF 103 +/- 2.4 20.3 +/- 1.3 19.7 3

## DTK 29.7 +/- 0.6 2 +/- 0 6.7 21

DH 31 75.3 +/- 3.2 4 +/- 0 5.3 3

Ast-C 36.8 +/-2 1.8 +/- 0.2 4.9 5

## Proctolin >400 24 +/- 0 <4 3

## *PTTH*-Gal4 4 +/- 0 0 +/- 0 0 3
